# Prevalence of Dyslipidemia and Management in the Thai Population, National Health Examination Survey IV, 2009

**DOI:** 10.1155/2014/249584

**Published:** 2014-03-30

**Authors:** Wichai Aekplakorn, Surasak Taneepanichskul, Pattapong Kessomboon, Virasakdi Chongsuvivatwong, Panwadee Putwatana, Piyamitr Sritara, Somkiat Sangwatanaroj, Suwat Chariyalertsak

**Affiliations:** ^1^Department of Community Medicine, Faculty of Medicine, Ramathibodi Hospital, Mahidol University, Rama VI Road, Rajdevi, Bangkok 10400, Thailand; ^2^National Health Examination Survey Office, Nonthaburi, Nonthaburi 11000, Thailand; ^3^College of Public Health Sciences, Chulalongkorn University, Bangkok 10330, Thailand; ^4^Faculty of Medicine, Khon Kaen University, Khon Kaen 40002, Thailand; ^5^Epidemiology Unit, Faculty of Medicine, Prince of Songkla University, Songkhla 90110, Thailand; ^6^Ramathibodi School of Nursing, Faculty of Medicine, Ramathibodi Hospital, Bangkok 10400, Thailand; ^7^Department of Medicine, Faculty of Medicine, Ramathibodi Hospital, Bangkok 10400, Thailand; ^8^Department of Medicine, Faculty of Medicine, Chulalongkorn University, Bangkok 10330, Thailand; ^9^Faculty of Medicine, Chiang Mai University, Chiang Mai 50002, Thailand

## Abstract

This study determined the prevalence and management of dyslipidemia in Thai adults using data from the Thai National Health Examination Survey IV in 2009. Dyslipidemia was defined based on the Third Adult Treatment Panel guidelines. A total of 19,021 adults aged 20 yr and over were included. Mean (SE) levels of total cholesterol, HDL-C, LDL-C, and triglycerides were 206.4 (1.03), 46.9 (0.34), 128.7 (1.09), and 131.4 (2.20) mg/dL, respectively. Prevalence of high LDL-C, low HDL-C, and high triglycerides were 29.6 %, 47.1 %, and 38.6%, respectively. Compared with individuals in the north and northeast, residents in Bangkok and Central region had significant higher levels of LDL-C but lower level of HDL-C. Triglyceride level was the highest in the northeast residents. Overall, 66.5% of Thais had some forms of dyslipidemia. Awareness and treatment of high LDL-C among those with high LDL-C were 17.8% and 11.7%, respectively. Among individuals aware of high LDL-C, those at highest CHD risk compared with those at low risk had higher percentage of treatment (73.1% versus 51.7%, resp.) but lower percentage of control at goal (32.9% versus 76.4%, resp.). Various forms of dyslipidemia are common in Thai adults, with a low level of awareness and treatment of high LDL-C.

## 1. Introduction

Association of dyslipidemia with the development of cardiovascular disease (CVD) is well established and guidelines for management of the condition have already been issued [1–5]. Serum total cholesterol other than its subtypes is usually used as a measure for monitoring at the population level, especially in developing countries. In the past few decades, data from several countries reported a high prevalence of dyslipidemia and unsatisfactory results of dyslipidemia management [6–12]. Recently, a multicountry analysis of national health examination survey data from eight countries on various continents reported disappointing findings of low detection and inadequate management of high serum cholesterol, particularly among middle-income countries [12]. Among these countries, Thailand in 2004 had the highest (78%) percentage of unawareness of hypercholesterolemia, with markedly low percentages of treatment and control. In clinical practice, low-density lipoprotein cholesterol (LDL-C) is usually the primary target for lipid management [1]. Recent studies suggest that low HDL-C and high triglycerides also confer residual risk for CVD [13, 14]. However, there is no Thai data on the prevalence of other lipid parameters—for example, high-density lipoprotein cholesterol (HDL-C), non-HDL-C, LDL-C, and triglycerides—in the general population, with and without CVD risk factors. Previous studies on the percentage of attaining LDL-C goals recommended by the Third Adult Treatment Panel (ATP III) cholesterol guidelines in 2001 [1] were based on hospital data [15–17]. The fourth National Health Examination Survey conducted in 2009 (NHES, 2009) was the first national survey in Thailand by which major lipid parameters including total cholesterol, HDL-C, and triglyceride were measured. The present study aimed to document the distribution of lipid parameters and prevalence of dyslipidemia among Thai adults aged ≥20 years old according to age, sex, area of residence, and geographic region. This report could provide useful information for prevention and control of dyslipidemia as well as baseline data for monitoring and evaluation at the national level.

## 2. Methods

The survey was approved by the Ethical Review Committee for Research in Human Subjects, Ministry of Public Health. The design of NHES 2009 was described elsewhere [18]. Briefly, it was based on a multistage probability sampling technique where sampling units in each of the four stages were (1) five provinces in each of the four main regions (north, northeast, central, and south) and Bangkok, (2) two to three districts in each selected province, (3) 13-14 enumeration units (EU)/villages in each district, and (4) individuals among six age groups (15–29, 30–44, 45–59, 60–69, 70–79, and ≥80 years) of each gender from each EU/village. The final sample size was 20,426 and the response rate was 93.1%. The present analysis omitted adolescents (age < 20 years), keeping 9,021 men and 10,000 women in the study. Urban area was defined by the Thai National Statistical Office as a municipal area in each province, including the entire Bangkok metropolis and officially designated sanitary district which is a locality achieving a minimum population size and density and income, and the remainders of the country were rural areas.

### 2.1. Data Collection and Measurement

Key variables included demographic data, behavioral risk factors, medical history of previously diagnosed diabetes, hypertension, and high blood cholesterol; and whether on medication. Blood pressure was measured using a standardized automatic blood pressure monitor (model A100; Microlife, Taipei, Taiwan). Each participant was seated for at least 5 min before the first reading of three serial measurements of blood pressure taken 1 min apart while in a sitting position. Venous blood samples were obtained from participants in the morning after fasting 12 h overnight. Plasma glucose was measured using a hexokinase enzymatic method. Serum samples were frozen and transferred to a central laboratory in Ramathibodi Hospital, Mahidol University, Bangkok. Serum total cholesterol and triglycerides were analyzed using enzymatic colorimetric methods (CHOD-PAP and GPO-PAP, resp.). High-density lipoprotein cholesterol (HDL-C) was measured using homogeneous enzymatic colorimetric method. Low-density lipoprotein cholesterol (LDL-C) was calculated based on the Friedewald formula [19] for subjects with triglycerides <4.5 mmol/L (400 mg/dL) and was directly measured by enzymatic method for those having triglycerides ≥4.5 mmol/L (400 mg/dL). All lipid measurements were carried out using a Hitachi 917 biochemistry analyzer (Roche Diagnostics, Basel, Switzerland). The laboratory was standardized according to the criteria of the Lipid Standardization Program of the Centers for Disease Control and Prevention and the National Heart, Lung, and Blood Institute [20].

### 2.2. Dyslipidemia

Recommended lipid levels were classified based on the guidelines of the Adult Treatment Panel III (ATP III) of the National Cholesterol Education Program [1, 2]. For estimation of coronary heart disease (CHD) risk, we used the World Health Organization (WHO) risk prediction charts for Southeast Asian countries (SEAR B) including Thailand to assign the 10-year CHD risk [21]. Variables used to categorize CHD risk included sex, age, diabetes status, systolic blood pressure, smoking status, and total cholesterol level. Low HDL-C was defined as HDL-C <1.03 mmol/L (40 mg/dL) in men and <1.30 mmol/L (50 mg/dL) in women. The cutoff points for high LDL-C were defined as follows: high risk, LDL-C ≥100 mg/dL if having prior CHD or CHD equivalent or having 10-year CHD risk >20%; moderate risk, LDL-C ≥130 mg/dL if having ≥2 risk factors (RF) and/or 10-year CHD risk 10% to 20%; and lower risk, LDL-C ≥160 mg/dL if having 0-1 RF. Other major independent risk factors, according to ATP III, include smoking, hypertension (systolic blood pressure ≥140 mmHg or diastolic blood pressure ≥90 mmHg or on antihypertensive medication), low HDL-C (<40 mg/dL), family history of premature CHD, or CHD in a first-degree relative (men <55 yrs and women <65 yrs), and age (men ≥45 yrs and women ≥55 yrs). If a person has high HDL-C (≥60 mg/dL), then one RF would be subtracted from the count. High non-HDL-C was defined based on the CHD risk similar to high LDL-C, with cutoff points for non-HDL-C of 190, 160 and 130 mg/dL corresponding to LDL-C levels of 160, and 130 and 100 mg/dL, respectively. Diabetes was defined as having a previous diagnosis of diabetes by a physician and currently taking hypoglycemic drugs during the prior two weeks, or those having fasting plasma glucose ≥7.0 mmol/L (126 mg/dL). Hypertension was defined as systolic blood pressure (SBP) ≥140 mmHg or diastolic blood pressure (DBP) ≥90 mmHg, or use of blood pressure-lowering medication. In order to explore the picture of isolated and combined abnormal lipid parameters, a mixed dyslipidemia condition was categorized into eight groups as follows: (1) isolated high LDL-C; (2) isolated low HDL-C; (3) combined high LDL-C and low HDL-C; (4) isolated high triglycerides (>2.26 mmol/L, 200  mg/dL); (5) combined high LDL-C and high triglycerides; (6) combined low HDL-C and high triglycerides; (7) combined high LDL-C, low HDL-C, and high triglycerides; and (8) no dyslipidemia.

For calculation of awareness, treatment and control of high LDL-C, subjects who answered “Yes” to the question “Have you ever been told by a health professional or physician that you had high blood cholesterol?” were considered to be aware of the condition. Undergoing treatment for high blood cholesterol was defined as an affirmative response to the question “Have you taken medication for lowering blood cholesterol in the past two weeks?” We calculated the percentage of individuals who were aware of the condition. The percentage of treatment was calculated for all participants with high LDL-C and for those aware of the condition. The percentage of controlled LDL-C was calculated for all participants with high LDL-C and for those receiving treatment.

### 2.3. Statistical Methods

Complex survey analysis was employed to take into account the probability sampling design. All lipid parameters were presented as age-adjusted arithmetic mean, except triglycerides for which geometric mean was calculated due to the skewed distribution. The age-adjusted prevalences of high LDL-C, low HDL-C, and high triglycerides were calculated overall and for subgroups according to age group, sex, area of residence, geographic region, and educational level, as well as for those with comorbidity of diabetes or hypertension and according to CHD risk categories. We also calculated the age-adjusted prevalence of mixed dyslipidemia for men and women. All comparisons were age- and sex-standardized to the national population in 2008. Adjusted Wald tests were used to examine the differences, with *P* < 0.05 considered statistically significant. We used linear regression to evaluate linear trend of each lipid parameter level by age groups and CHD risk stratification, and reported the *P* value for trend. For evaluation of trends in prevalence of each lipid abnormality by age group and CHD risk, logistic regression model was used. All of the analyses were performed using Stata statistical software version 10 (StataCorp, College Station TX, USA).

## 3. Results

Table 1 shows the age-adjusted means (SE) of total cholesterol, HDL-C, non-HDL-C and LDL-C, total cholesterol to HDL-C ratio (TC/HDL-C) and geometric mean of triglycerides among Thai adults aged ≥20 yrs. Overall, the age-adjusted means of total cholesterol, HDL-C, non-HDL-C, and LDL-C (but not triglycerides and TC/HDL-C) were higher in women than in men (all *P* < 0.001) as well as in urban areas compared with rural areas (all *P* < 0.01). All lipid levels, except HDL-C, were higher in men of middle age (35–59 yrs) and in women of older age (≥60 yrs). There were slight differences in lipid parameters according to region. Individuals who resided in Bangkok, south and central regions, had higher levels of LDL-C, non-HDL-C, and HDL-C but had lower levels of triglycerides than those living in the northern and northeastern regions. Individuals with diabetes or hypertension had a markedly higher level of triglycerides than those without these conditions (all *P* < 0.01). Those with higher CHD risk were more likely to have unfavorable levels of HDL-C and triglycerides compared with those categorized as low CHD risk.

**Figure 1:**
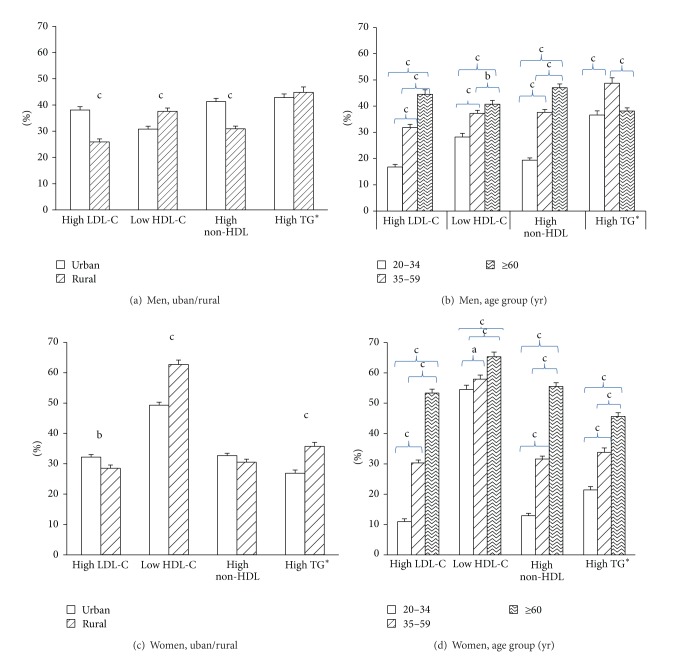
Age-adjusted prevalence of dyslipidemia by area of residence ((a) and (c)) and age-specific prevalence of dyslipidemia ((b) and (d)) among Thai men and women aged ≥20 years, NHES IV 2009. Upper error bars indicate SE values. Significant level: a, *P* < 0.05; b, *P* < 0.01; c, *P* < 0.001.

Figure 1 displays the age-adjusted prevalence of dyslipidemia by area of residence and sex. Overall, the age-adjusted prevalence of high LDL-C and of high non-HDL-C were similar for men and women. Women had higher prevalence of low HDL-C but lower prevalence of high triglycerides compared with men (*P* < 0.01). Urban residents had higher prevalence of both high LDL-C and high non-HDL-C, whereas rural residents had higher prevalence of low HDL-C. For high triglycerides, the prevalence in urban/rural areas differed by sex; there was no significant difference between urban and rural areas among men, but the prevalence was higher for women in rural areas compared with their counterparts in urban areas.

**Figure 2:**
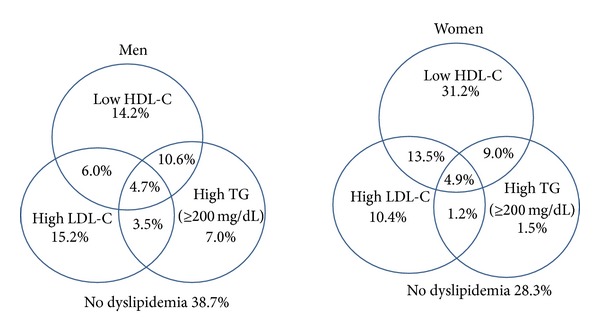
Age-adjusted prevalence of each lipid abnormality and mixed dyslipidemia in Thai men and women aged ≥20 years, NHES IV 2009.

Figure 2 shows the age-adjusted prevalence of dyslipidemia for each parameter and the combined lipid parameters for men and women. Overall, 22.2% had isolated low HDL-C (14.2% in men versus 31.2% in women, *P* < 0.05), 12.8% had isolated high LDL-C (15.2% in men versus 10.4% in women), 4.2% had high triglycerides (7% in men versus 1.5% in women), and 4.8% had a combination of three lipid abnormalities (4.7% in men versus 4.9% in women). About 6% of men and 13.5% of women had combined low HDL-C and high LDL-C, while 10.6% of men and 9% of women had combined low HDL-C and high triglycerides. The prevalence of high LDL-C combined with high triglycerides was 3.5% in men and 1.2% in women. Thus, 33.5% of Thai adults had no dyslipidemia (38.7% of men and 28.3% of women).

Table 2 shows that people in the northeast tended to have higher prevalence of high triglycerides and low HDL-C, whereas people in the south and Bangkok tended to have higher prevalence of high LDL-C but lower prevalence of low HDL-C. Individuals with diabetes or hypertension had higher percentages of dyslipidemia including high LDL-C and high triglycerides. Men with diabetes and women with hypertension were more likely to have higher prevalence of low HDL-C compared with those without the conditions.

Table 3 shows the age-adjusted prevalence of awareness, treatment, and control of high LDL-C. Overall, 17.8% of those with high LDL-C were aware that they had high LDL-C, and the percentages of treated and controlled among all individuals with high LDL-C were markedly low (11.7 and 6.3%, resp.). However, among those aware of their condition, 60.6% overall were treated; of those treated, 57.6% had LDL-C at recommended levels. Low percentages of awareness and treatment were observed for all characteristics; the percentages were slightly higher among individuals in urban areas compared with those in rural areas and also were higher in Bangkok compared with other regions. Note that the percentages of treatment among those aware of their diagnosis were higher for those with diabetes, hypertension, or high risk of CHD than those without these conditions; however, the percentages of controlled among those treated were the opposite.

## 4. Discussion

The present study demonstrated a high prevalence of dyslipidemia in the Thai population. There were significantly different patterns of lipid abnormality by sex, urban/rural areas, and geographic regions. High LDL-C was more prevalent in individuals residing in urban areas than in rural areas, and also was more common in Bangkok, the south and central regions, than in the north and northeast, where people were more likely to have lower HDL-C and high triglyceride levels. The prevalence of dyslipidemia was also worse among those with comorbidity of diabetes, hypertension, and increased CHD risk. The present study also revealed that 8 in 10 of the individuals with high LDL-C remained unaware and not treated.

Compared with the lipid levels of the U.S. population in 2007–2010, Thai people have higher levels of TC (206 versus 196 mg/dL), LDL-C (128.7 versus 116 mg/dL), non-HDL-C (158.5 versus 144 mg/dL), and triglycerides (geometric mean, 131.45 versus 110 mg/dL) but lower HDL-C (46.93 versus 52.5 mg/dL) [7, 22]. The current cholesterol levels of the Thai population are comparable to the levels in the U.S. in 1988–1994 [23]. The level of HDL-C in the Thai population is slightly lower than that of the Korean population reported in 2006 and 2012 [9, 24]. The Korea health survey reported that the levels of TC, LDL-C, HDL-C, and triglycerides were 184.7, 114, 45.2, and 135.2 mg/dL, respectively, which were more favorable than what was found in the Thai population in the present study [9].

The findings of slightly higher levels of total cholesterol (TC) and LDL-C but lower level of triglycerides in women compared with men were consistent with a previous study [25] as well as other studies in Japan [26], Korea [27], and the U.S. [23]; however, the magnitudes of differences were smaller among the high-income countries. In the present study, the finding of higher TC and LDL-C in women is concordant with the higher prevalence of obesity and abdominal obesity in Thai women than in men [28]. Obesity and abdominal obesity affect lipid metabolism and contribute to dyslipidemia, whereas body weight reduction improves lipid abnormalities [3, 29].

The findings of high prevalence of dyslipidemia in Thai population might not be surprised as dietary pattern in the current Thai food has gradually changed from the past. The traditional Thai food normally constitutes low fat, high complex carbohydrate, and high fiber as compared with westernized food [30]. During the past several decades since 1960, the per capita consumption of cereals and tuber, particularly rice, has declined considerably compared with the early 1960 [31–33]. Consumption of animal products increased after 1975 with dramatic increases in 1985 [30, 33]. The changes in dietary pattern owing to the economic development and urbanization are likely to contribute to the current situation of dyslipidemia. Compared with the findings from a previous study in 2000 in five provinces [25], the levels of TC, LDL-C, non HDL-C, and triglycerides in the present study were higher; however, the pattern of differences across regions was relatively similar, as TC, LDL-C, and HDL-C remained higher in Bangkok and the central region, whereas people in the northeast had the lowest level of HDL-C and highest level of triglycerides. The different type of lipid abnormality is likely to be attributed to the different food pattern. The higher levels of TC, LDL-C, and HDL-C are likely to be related to urbanization, where people consume more meat and a fattier diet. People in the rural northeast usually consume more carbohydrates in the form of sticky rice. Men in rural areas also consume greater amounts of alcohol and carbohydrates but less protein and fat in their daily diet, which might contribute to the higher levels of triglycerides and lower cholesterol among the population in this region. Data from the Thai consumption survey reported that average daily consumptions of protein, fat, and carbohydrate were 72, 60, and 311 gram per person, respectively. Fat consumption was higher among urban household and carbohydrate consumption was higher in the rural areas [34]. People in the northeast had the lowest amount of fat consumption [34]. Compared to other regions, the proportions of energy intake from carbohydrate among northeast residents were the highest at 59.2% and from fat were the lowest at 23.3%, whereas the corresponding percentages for people in Bangkok were at 54.4% and 31.4%, respectively [32]. It is unlikely that the higher consumption of carbohydrate among the rural residents was due to the higher percentages of vegetarians in rural areas; however, this issue needs further investigation. With regard to the effect of high carbohydrate intake, a meta-analysis of 60 controlled studies showed that high dietary carbohydrate intake increased fasting triglyceride level and decreased HDL-C [35]. In addition, consumption of high glycemic index and glycemic load food has been reported to be associated with CHD [36]. However, research into the link of dietary pattern and other behavioral and environment factors with lipid profiles and health outcomes by region needs further investigation.

Another important finding in the present study is the markedly low percentage of awareness and treatment of LDL in the population. The overall control percentage among those treated was relatively moderate (58%) and the percentages of attaining LDL-C targets were comparable to the findings from other studies in clinical settings [15, 16]; however, this should be interpreted with caution, given the difference in sample characteristics, time of study, and tools to estimate CHD risk. In the U.S. study, the percentage of awareness of high LDL-C was 61.5%, treatment among those aware was 70%, and control among those treated was 63.6% [37]. The corresponding percentage for awareness in the present study was about 3.4 times lower, but percentages for treatment and control among those treated were only slightly lower. This finding might reflect that the effort to achieve a higher screening rate for detection of high serum cholesterol was more difficult than the effort to deliver treatment and control for those treated for hypercholesterolemia, which is consistent with a previous study [12].

Although Thailand has a good infrastructure of district health hospitals distributed throughout the country, the shortage of manpower and laboratory facilities to screen and monitor serum cholesterol and type of cholesterol remains a problem, particularly in rural areas. This is shown by the even lower percentages in rural areas compared with urban areas and lower percentages in the peripheral regions as compared with Bangkok. Since the previous decade, the Ministry of Public Health has spent a great deal of effort in an attempt to increase the coverage in screening for diabetes and hypertension, due to the higher burden of hypertension and diabetes compared to hypercholesterolemia in the Thai population [38]; however, for dyslipidemia, more resources are needed for investment in rural health care services. A study reported that Thailand had invested less efficiently compared with Mexico in terms of provision of medication [39]. Further intervention for efficient allocation of technical resources and workforce is required to scale up the facilities for screening programs and therapeutic lifestyle changes. It should be noted that when comparing to those without diabetes, hypertension, or moderate/high CHD risk, the percentages of awareness and treatment among those with the conditions were higher. This might be due to the fact that they were patients in the health service system, so the conditions were more likely to be detected and treated with medication. However, the findings of worse control among these groups are consistent with other studies [15–17, 37]. Since guidelines in the management of dyslipidemia are available [4, 5], further efforts are needed to improve LDL-C control in those with diagnosed high LDL-C, particularly for those at high risk of CHD.

Some limitations in the present study need to be mentioned. Measurement of lipids for each participant was done at a single point in time, and data on type of medication is not known. The estimation of CHD risk was based on an international WHO prediction score. The estimation might misclassify the risk category; however, we used a Thai coronary heart risk score to estimate the risk and the findings did not change substantially [40]. Treatment information was based on self-reports and did not include lifestyle modification or other herbal medication. Despite the limitations, the present study is the first national representative population-based study with a large sample size and provides more detailed information on dyslipidemia in Thai adults. The use of the WHO prediction chart for estimation of CHD risk should provide more useful information to stratify individuals' risk. In conclusion, dyslipidemia is a common condition with various forms in Thai adults. Effective intervention to promote healthy dietary intake and increased physical activity should be intensified at the population level. Appropriate screening, treatment, and therapeutic lifestyle change programs for high-risk groups must be scaled up.

## Figures and Tables

**Table 1:** Age-adjusted mean total cholesterol, HDL-C, non-HDL-C, LDL-C, total cholesterol to HDL-C ratio, and geometric mean triglycerides by age group, area of residence, region, comorbidity, and CHD risk among Thai adults aged ≥20 years, NHES 2009.

	*n*	Total cholesterol	HDL-C	Non-HDL-C	LDL-C	Triglycerides	TC/HDL-C
		Mean (SE)	Mean (SE)	Mean (SE)	Mean (SE)	Geometric mean (SE)	Mean (SE)
Both sexes	19,021	206.4 (1.03)	46.9 (0.3)	159.5 (0.8)	128.7 (1.1)	131.4 (2.2)	4.6 (0.02)
Age group							
20–34	2,589	196.4 (1.0)^c^	48.2 (0.30)^c^	148.2 (0.9)^c^	121.6 (1.03)^c^	112.2 (1.9)^c^	4.2 (0.02)^b^
35–59	7,356	209.2 (1.2)	47.0 (0.4)	162.2 (0.9)	130.3 (1.2)	136.5 (2.7)	4.7 (0.03)
≥60	9,076	212.9 (1.4)	45.5 (.04)	167.4 (1.1)	135.2 (1.2)	141.6 (1.9)	4.9 (0.03)
Area of residence							
Urban	10,327	213.4 (0.9)^b^	48.7 (0.3)^b^	164.7 (0.8)^b^	135.3 (1.0)^b^	124.3 (2.3)^b^	4.6 (0.03)
Rural	8,689	203.4 (1.2)	46.1 (0.4)	157.3 (1.0)	125.8 (1.3)	134.8 (2.5)	4.6 (0.02)
Region							
North	4,335	198.3 (2.3)^c^	46.9 (0.5)^b^	151.4 (2.0)^c^	122.4 (1.8)^c^	125.2 (1.8)^c^	4.4 (0.04)^a^
Central	4,549	215.7 (1.4)	48.4 (0.5)	167.3 (1.4)	136.8 (1.7)	129.6 (2.4)^c^	4.7 (0.04)
Northeast	4,191	197.4 (1.0)^c^	44.6 (0.5)^c^	152.8 (0.8)^c^	118.6 (1.1)^c^	147.5 (4.3)^c^	4.6 (0.04)
South	3,971	216.8 (1.1)	47.8 (0.5)^a^	169.1 (1.2)	141.5 (1.3)	119.1 (1.5)	4.8 (0.05)^a^
Bangkok	1,975	216.4 (0.9)	49.8 (0.5)	166.6 (1.1)	139.3 (1.03)	114.4 (1.6)	4.6 (0.05)
Diabetes							
Yes	2,048	209.4 (2.0)	44.3 (0.3)^b^	165.1 (1.9)^b^	124.5 (1.7)^a^	178.2 (4.6)^b^	5.0 (0.05)^b^
No	16,441	206.3 (1.1)	47.2 (0.3)	159.2 (0.9)	129.1 (1.1)	128.9 (2.2)	4.6 (0.02)
Hypertension							
Yes	6,596	214.3 (1.3)^b^	46.4 (0.3)	167.9 (1.4)^b^	131.7 (1.3)^b^	158.6 (3.03)^b^	4.8 (0.04)^b^
No	12,406	204.2 (1.1)	46.9 (0.4)	157.3 (0.9)	127.8 (1.2)	126.3 (2.3)	4.6 (0.02)
CHD risk stratification							
0-1 RF	9,477	209.4 (1.0)^c^	51.0 (0.4)^c^	158.5 (0.8)^c^	131.7 (1.1)	116.8 (1.9)^c^	4.2 (0.02)^c^
2+ RF and/or 10-year risk 10–20%	6,066	194.9 (1.5)	38.4 (0.2)	156.5 (1.4)	118.9 (1.4)	164.8 (3.3)	5.2 (0.04)
CHD risk equivalent (10-year risk >20%)	3,224	213.6 (1.6)	45.1 (0.4)	168.5 (1.5)	130.1 (1.4)	165.8 (4.2)	4.9 (0.04)
Men	9,021	203.0 (1.2)^c^	45.2 (0.3)^c^	157.9 (1.0)^b^	124.3 (1.2)^c^	143.3 (2.6)^c^	4.7 (0.02)^b^
Age group							
20–34	1,268	196.6 (1.2)^c^	46.4 (0.4)^c^	149.2 (1.0)^c^	119.2 (1.2)^c^	127.1 (2.6)^c^	4.4 (0.03)^c^
35–59	3,300	206.0 (1.3)	44.9 (0.3)	161.1 (1.1)	125.3 (1.3)	152.5 (3.3)	4.8 (0.03)
≥60	4,453	203.3 (1.7)	44.2 (0.5)	159.1 (1.3)	128.5 (1.5)	135.4 (2.0)	4.8 (0.03)
Area of residence							
Urban	4,755	212.7 (1.5)^b^	46.5 (0.4)^b^	166.2 (1.3)^c^	133.2 (1.6)^c^	140.0 (2.9)	4.8 (0.03)^a^
Rural	4,266	199.0 (1.2)	44.6 (0.3)	154.4 (1.1)	120.5 (1.3)	144.8 (3.1)	4.7 (0.03)
Region							
North	2,103	194.5 (2.4)^c^	45.4 (0.5)^a^	149.1 (2.1)^c^	117.5 (2.2)^c^	135.9 (2.4)	4.5 (0.04)^c^
Central	2,201	213.0 (1.6)^a^	46.0 (0.4)	167.0 (1.5)	133.0 (1.9)^b^	143.7 (3.0)^b^	4.9 (0.04)
Northeast	2,050	191.9 (1.3)^c^	43.1 (0.5)^c^	148.8 (1.3)^c^	112.1 (1.2)^c^	158.7 (5.6)^c^	4.6 (0.1)
South	1,891	214.9 (1.5)	43.6 (0.5)	168.3 (1.5)	138.9 (1.5)	127.0 (2.7)	4.8 (0.05)
Bangkok	776	219.5 (1.9)	47.7 (0.7)	171.8 (2.0)	141.1 (1.9)	128.5 (3.2)	4.8 (0.1)
Diabetes							
Yes	890	201.1 (3.1)	40.6 (0.5)^a^	160.4 (3.1)	116.4 (2.5)^b^	187.3 (8.0)^c^	5.2 (0.1)^b^
No	7,857	202.8 (1.2)	45.4 (0.3)	157.4 (1.0)	124.5 (1.3)	141.0 (2.7)	4.7 (0.02)
Hypertension							
Yes	3,198	232.9 (11.9)^c^	46.1 (0.4)^b^	166.6 (1.6)^c^	127.7 (1.6)^a^	169.2 (3.6)^c^	4.9 (0.05)^b^
No	5,809	167.7 (3.1)	44.9 (0.3)	155.7 (1.0)	123.4 (1.3)	137.8 (2.8)	4.7 (0.02)
CHD risk stratification							
0-1 RF	3,509	208.1 (1.1)	50.9 (0.4)^c^	157.2 (1.0)^c^	128.9 (1.2)^a^	122.6 (2.1)^c^	4.2 (0.02)^c^
2+ RF and/or 10-year risk 10–20%	3,871	195.8 (1.7)	38.5 (0.3)	157.4 (1.6)	118.6 (1.6)	168.8 (3.6)	5.3 (0.04)
CHD risk equivalent (10-year risk >20%)	1,465	206.8 (2.7)	42.3(0.5)	164.5 (2.7)	122.4 (2.6)	178.6 (5.8)	5.1 (0.1)
Women	10,000	209.8 (1.0)	48.7 (0.4)	161.1 (0.9)	133.2 (1.1)	120.5 (2.1)	4.5 (0.03)
Age group							
20–34	1,321	197.1 (1.1)^c^	50. 1 (0.3)^c^	147.0 (1.0)^c^	124.1 (1.0)^c^	98.6 (1.6)^c^	4.1 (0.03)^c^
35–59	4,056	212.1 (1.2)	48.9 (0.4)	163.3 (0.9)	134.9 (1.2)	123.2 (2.5)	4.5 (0.03)
≥60	4,623	220.7 (1.4)	46.6 (0.4)	174.1 (1.2)	140.7 (1.3)	146.7 (2.1)	4.9 (0.04)
Area of residence							
Urban	5,572	214.2 (0.8)^b^	51.0 (0.3)^b^	163.2 (0.8)^b^	137.4 (0.8)^c^	110.3 (2.1)^c^	4.4 (0.03)^b^
Rural	4,423	207.8 (1.3)	47.6 (0.4)	160.1 (1.1)	131.2 (1.4)	125.5 (2.4)	4.5 (0.03)
Region							
North	2,232	202.0 (2.4)^c^	48.3 (0.6)^c^	153.8 (2.1)^b^	127.2 (1.6)^c^	115.3 (3.2)^c^	4.3 (0.05)
Central	2,348	218.5 (1.3)^b^	50.7 (0.7)	167.7 (1.4)^c^	140.6 (1.7)	116.9 (2.3)^c^	4.5 (0.05)^b^
Northeast	2,141	202.9 (1.4)^c^	46.2 (0.5)^c^	156.7 (1.0)^b^	125.2 (1.6)^c^	137.1 (3.5)^c^	4.6 (0.03)^c^
South	2,080	218.8 (1.4)^b^	48.9 (0.6)^c^	169.8 (1.5)^c^	144.1 (1.4)^c^	111.6 (1.6)^c^	4.6 (0.1)^c^
Bangkok	1,199	213.2 (0.7)	51.9 (0.4)	161.3 (0.7)	137.5 (0.6)	101.9 (1.3)	4.3 (0.03)
Diabetes							
Yes	1,158	217.8 (2.6)^a^	47.9 (0.5)	169.9 (2.5)^b^	132.6 (2.2)	169.5 (5.1)^c^	4.7 (0.05)^b^
No	8,584	209.9 (1.1)	48.9 (0.4)	161.0 (0.9)	133.7 (1.1)	117.9 (2.1)	4.5 (0.03)
Hypertension							
Yes	3,398	191.9 (6.0)^c^	46.7 (0.4)^c^	169.2 (1.5)^c^	135.7 (1.4)^a^	148.7 (3.6)^c^	4.8 (0.05)^b^
No	6,597	137.0 (2.6)	48.8 (0.4)	159.0 (1.0)	132.3 (1.1)	115.8 (2.1)	4.4 (0.03)
CHD risk stratification							
0-1 RF	5,968	210.8 (1.0)^c^	51.1 (0.4)^c^	159.8 (0.9)^c^	134.4 (1.1)^c^	111.4 (2.1)^c^	4.3 (0.03)^c^
2+ RF and/or 10-year risk 10–20%	2,195	193.9 (2.0)	38.3 (0.3)	155.6 (2.0)	119.2 (2.0)	160.9 (5.3)	5.2 (0.1)
CHD risk equivalent (10-year risk >20%)	1,759	220/3 (1.9)	47.9 (0.5)	172.4 (1.8)	137.9 (1.6)	153.9 (4.8)	4.7 (0.04)

Significant level: ^a^ *P* < 0.05; ^b^ *P* < 0.01; ^c^ *P* < 0.001.

*P* values for comparison among age groups and CHD risk stratification were *P* for trend.

For comparison among regions: each region was compared with Bangkok.

**Table 2:** Age-adjusted prevalence of high total cholesterol, high LDL-C, low HDL-C, high non-HDL-C, and high triglycerides by age group, area of residence, region, comorbidity, and CHD risk among Thai men and women aged ≥20 years, NHES 2009.

	*n*	% High LDL-C*	% Low HDL-C (<40/50)	% High non-HDL-C	% High triglycerides (≥150)
Both sexes	19,021	29.6 (0.9)	47.1 (1.0)	32.6 (0.8)	38.6 (1.4)
Men	9,021	29.5 (1.0)	35.6 (1.1)^c^	34.0 (0.9)^b^	44.3 (1.7)^c^
Women	10,000	29.7 (0.9)	58.6 (1.2)	31.2 (0.8)	32.9 (1.2)
*Men *					
Region					
North	2,103	22.6 (2.0)^c^	35.1 (1.8)^b^	26.3 (1.9)^c^	41.7 (1.3)^a^
Central	2,201	38.2 (1.5)	32.5 (1.0)^b^	42.1 (1.5)	42.4 (1.9)^a^
Northeast	2,050	19.3 (0.9)^c^	42.9 (1.9)^c^	26.2 (1.3)^c^	52.5 (3.6)^c^
South	1,891	42.0 (1.5)	29.4 (0.9)	44.2 (1.4)	33.4 (1.8)
Bangkok	776	42.2 (2.4)	26.5 (1.4)	44.0 (2.4)	36.9 (0.9)
Diabetes					
Yes	890	66.6 (2.8)^c^	54.6 (3.1)^c^	79.7 (2.4)^c^	55.3 (1.4)^c^
No	7,857	28.0 (1.1)	34.2 (1.1)	32.1 (0.9)	41.4 (2.0)
Hypertension					
Yes	3,198	39.1 (1.7)^c^	35.3 (1.3)	47.9 (1.7)^c^	55.3 (1.4)^c^
No	5,809	26.4 (0.9)	35.9 (1.2)	29.7 (0.8)	41.4 (2.0)
CHD risk stratification					
0-1 RF	3,509	19.4 (1.0)^c^	9.4 (0.5)^c^	19.3 (0.8)^c^	32.8 (1.5)^c^
2+ RF and/or 10-year risk 10–20%	3,871	35.1 (1.8)	72.1 (1.3)	44.5 (1.7)	54.3 (2.2)
CHD risk equivalent (10-year risk >20%)	1,465	70.8 (2.7)	47.9 (2.8)	80.5 (2.0)	59.6 (2.7)
*Women *					
Region					
North	2,232	23.3 (1.9)^c^	60.4 (2.2)^c^	24.7 (1.9)^b^	30.5 (1.8)^c^
Central	2,348	34.8 (2.0)	50.2 (2.6)	35.8 (1.6)^a^	29.7 (1.5)^c^
Northeast	2,141	25.5 (1.0)^c^	66.8 (1.6)^c^	29.1 (1.1)	41.4 (1.9)^c^
South	2,080	38.2 (1.0)^c^	58.3 (2.6)^b^	37.0 (1.7)^a^	28.1 (1.0)^c^
Bangkok	1,199	32.5 (1.0)	47.4 (1.2)	31.8 (0.8)	22.6 (0.8)
Diabetes					
Yes	1,158	73.4 (2.5)^c^	59.6 (2.7)	78.0 (2.4)^c^	47.6 (2.6)^c^
No	8,584	27.0 (0.9)	57.8 (1.3)	28.1 (0.8)	30.2 (1.3)
Hypertension					
Yes	3,398	42.0 (1.5)^c^	66.0 (1.5)^c^	45.4 (1.6)^c^	47.6 (2.6)^c^
No	6,597	25.2 (0.9)	58.0 (1.3)	26.0 (0.9)	30.2 (1.3)
CHD risk stratification					
0-1 RF	5,968	20.8 (0.8)^c^	51.4 (1.3)^c^	20.6 (0.7)^c^	26.6 (1.2)^c^
2+ RF and/or 10-year risk 10–20%	2,195	34.7 (2.0)	90.2 (0.9)	39.8 (2.1)	54.9 (3.5)
CHD risk equivalent (10-year risk >20%)	1,759	76.5 (1.9)	58.8 (2.5)	(1.6)	55.2 (3.1)

*High LDL-C is based on ATP III classification, in which the CHD risk was calculated from the WHO CVD risk chart.

Significant level: ^a^ *P* < 0.05; ^b^ *P* < 0.01; ^c^ *P* < 0.001.

*P*values for comparison among age groups and among CHD risk stratification were *P*-for trend

For comparison among regions: each region was compared with Bangkok.

**Table 3:** Age-adjusted proportions of awareness, treatment, and control of high LDL-C among Thai adults aged ≥20 years with high LDL-C.

	% Aware	% Treated among high LDL-C	% Treated among aware of high LDL-C	% Control among high LDL-C	% LDL-C at recommended level among treated
Total	17.8 (1.1)	11.7 (0.8)	60.6 (1.5)	6.3 (0.5)	57.6 (2.7)
Sex					
Men	14.3 (1.2)^b^	9.2 (0.9)^c^	56.1 (2.4)^a^	4.5 (0.5)^c^	54.0 (3.4)
Women	21.2 (1.2)	14.2 (0.9)	65.1 (2.2)	8.2 (0.7)	61.2 (2.9)
Age					
20–34	5.6 (1.2)^c^	3.9 (0.9)^c^	54.9 (7.9)^c^	2.7 (0.6)^c^	43.2 (3.3)^b^
35–59	18.5 (1.0)	11.0 (0.7)	58.0 (2.4)	6.4 (0.5)	60.0 (2.7)
≥60	27.3 (1.8)	21.5 (1.6)	79.0 (1.5)	9.6 (0.4)	44.1 (2.8)
Area of residence					
Urban	24.2 (1.2)^c^	16.0 (0.9)^c^	63.8 (2.0)	7.4 (0.3)^a^	46.8 (1.9)^c^
Rural	13.8 (0.9)	9.0 (0.7)	61.3 (2.8)	5.6 (0.6)	66.6 (4.3)
Region					
North	15.7 (1.1)^c^	10.1 (0.7)^c^	58.6 (4.1)	6.3 (0.8)	63.0 (8.0)
Central	16.1 (0.8)^c^	11.2 (0.7)^c^	61.3 (3.4)	5.8 (0.4)^b^	56.3 (3.4)
Northeast	14.2 (2.3)^c^	9.7 (1.8)^c^	64.4 (2.9)	6.8 (1.5)	69.0 (2.3)^c^
South	18.3 (0.8)^c^	11.2 (0.7)^c^	60.7 (4.0)	5.5 (0.5)^b^	43.4 (3.3)
Bangkok	30.1 (1.1)	19.8 (1.2)	59.8 (2.9)	8.2 (0.6)	48.8 (3.5)
Diabetes					
Yes	27.4 (2.3)^c^	21.3 (1.8)^c^	76.3 (4.5)^b^	8.1 (1.4)	39.7 (2.6)^c^
No	15.5 (1.0)	9.5 (0.7)	56.9 (2.1)	5.8 (0.4)	62.7 (2.9)
Hypertension					
Yes	25.5 (1.5)^c^	17.8 (1.3)^c^	70.0 (3.0)^c^	9.3 (0.7)^c^	54.6 (3.3)
No	13.0 (0.9)	7.5 (0.6)	53.5 (2.0)	4.5 (0.4)	61.8 (2.7)
CHD risk stratification					
0-1 RF	14.8 (1.1)^c^	8.6 (0.8)^c^	51.7 (2.3)^c^	6.4 (0.6)	76.4 (3.2)^c^
2+ RF and/or 10-year risk 10–20%	19.2 (2.3)	15.2 (2.2)	73.3 (1.7)	11.2 (2.3)	60.5 (4.5)
CHD risk equivalent (10-year risk >20%)	25.5 (2.5)	19.3 (2.0)	73.1 (4.0)	5.9 (0.9)	32.9 (3.8)

Significant level: ^a^ *P* < 0.05; ^b^ *P* < 0.01; ^c^ *P* < 0.001.

*P* values for comparison among age groups and among CHD risk stratification were *P*-for trend.

For comparison among regions: each region was compared with Bangkok.
